# Fatigue in Inactive Auto-Inflammatory Diseases and Opportunities for Optimizing Clinical Care: A Single-Center Observational Study

**DOI:** 10.3390/jcm14238268

**Published:** 2025-11-21

**Authors:** Yilmaz Satirer, Özlem Satirer, Susanne M. Benseler, Jasmin B. Kuemmerle-Deschner

**Affiliations:** 1 Division of Pediatric Rheumatology, Department of Pediatrics and Autoinflammation Reference Center Tuebingen (arcT), University Hospital Tuebingen, 72076 Tuebingen, Germany; 2 Department of Pediatrics, Kreiskliniken Reutlingen, 72764 Reutlingen, Germany; 3 European Reference Network—Rare Immunodeficiency, Autoinflammatory and Autoimmune Disease (ERN-RITA);; 4 Department of Pediatrics, Cumming School of Medicine, University of Calgary, Calgary, AB T2N 1N4, Canada; 5 University College Dublin, D04 V1W8 Dublin, Ireland; 6 Trinity College Dublin, D02 F6N2 Dublin, Ireland; 7 Royal College of Physicians and Surgeons of Ireland, D02 YN77 Dublin, Ireland; 8 Dublin City University, D09 V209 Dublin, Ireland; 9 Children’s Health Ireland, D01 R5P3 Dublin, Ireland

**Keywords:** autoinflammatory diseases, CAPS, FMF, fatigue, depression, PedsQL-MFS, CES-D

## Abstract

**Objective:** To characterize debilitating fatigue in children and adults across inactive auto-inflammatory diseases (AID), identifying distinct disease-specific fatigue phenotypes and modifiable risk factors is necessary for optimal care. **Methods:** A single-center cohort of consecutive patients with inactive AID between 2007 and 2024 was performed. Demographics, clinical and laboratory features, and treatment were captured. Fatigue was characterized and quantified using the PedsQL-MFS and VAS; the CES-D/CESD-R was applied to assess depression risk. Comparisons were made using non-parametric methods, multivariable regression identified risk factors of fatigue in inactive disease. **Results:** 66 patients were included: 39 (59%) were children; the median age at symptom onset was 4 years, at treatment start was 8 years, and study follow-up was 7 years. All patients had inactive disease at the last visit. Patients with cryopyrin-associated periodic syndromes (CAPS) had the highest Cognitive Fatigue scores (*p* = 0.04). Univariate analyses identified higher fatigue scores (1) in adults across all domains except Sleep/Rest (all *p* ≤ 0.002), (2) in patients with pathogenic/likely pathogenic variants, and (3) for disease duration ≥10 years except Sleep/Rest (all *p* ≤ 0.01). Depression was the single most important factor associated with fatigue in all domains (*p* < 0.001). In multivariable analysis, depression remained the strongest predictor of fatigue even when accounting for age, gene variant, disease duration, and treatment delay. **Conclusions:** Fatigue remains the major burden in AID despite the availability of effective anti-inflammatory therapies. Depression was identified as the strongest determinant of debilitating fatigue in inactive AID. Systematic screening and integrated approaches addressing both psychological and inflammatory domains are essential for optimal care.

## 1. Introduction

Auto-inflammatory diseases (AID) constitute a group of rare, hereditary disorders characterized by dysregulation of the innate immune system, leading to chronic systemic inflammation [1]. Pro-inflammatory cytokines, particularly interleukin-1 (IL-1), play a pivotal role in driving inflammatory cascades that result in recurrent clinical manifestations such as fever, rash, arthralgia, and, notably, fatigue [1,2,3]. The advent of IL-1-targeted therapies have revolutionized disease management, effectively controlled systemic inflammation, and ameliorated many clinical features [2,4,5].

Fatigue, however, has emerged as one of the most pervasive and disabling symptoms in AID. Unlike ordinary tiredness, it is severe, persistent, and disproportionate to exertion or rest, substantially impairing quality of life and daily functioning [6]. Importantly, fatigue often persists even when systemic inflammation is adequately controlled by IL-1 blockade, suggesting that its etiology is complex and extends beyond direct inflammatory activity [7,8]. This residual fatigue contributes to long-term disability and diminished productivity. It results in a considerable societal burden [8].

In parallel, depressive symptoms have increasingly been recognized in patients with AID; they often remain underdiagnosed and undertreated. Chronic inflammation has been implicated in the pathogenesis of depression, with cytokines such as IL-1, IL-6, and TNF-α linked to altered mood regulation, neuroplasticity, and hypothalamic–pituitary–adrenal axis function [9]. Moreover, the psychosocial consequences of living with a chronic, unpredictable illness further heighten vulnerability to depression and other mental health disorders [10]. Depression in this context not only worsens overall disease burden but also interferes with adherence, functional capacity, and quality of life [7,10,11].

Despite the high prevalence of fatigue and depression in AID, their interrelationship has not been systematically examined to date. This knowledge gap hinders comprehensive patient management. It underscores the need for studies that characterize both symptoms, explore their correlation, and identify disease- and patient-related factors that contribute to their persistence.

Therefore, the aims of this study were as follows: (1) to characterize fatigue and depression in a longitudinal cohort of pediatric and adult patients with AIDs on effective therapy including global measures of fatigue severity, (2) to compare Overall Fatigue and its domains and depression across diseases, and (3) to identify modifiable risk factors associated with debilitating fatigue in patients with treated auto-inflammatory diseases.

## 2. Materials and Methods

A single-center cohort study of consecutive pediatric and adult patients with a confirmed diagnosis of an auto-inflammatory disease, including CAPS and Familial Mediterranean Fever (FMF), between January 2007 and June 2024 was performed. The inclusion criteria were as follows: (1) an established diagnosis of CAPS or FMF according to published criteria [1,4,12]; (2) age ≥ 8 years at the last visit [13], and (3) evidence of inactive disease either on or off treatment at the last follow-up visit [14]. Patients with comorbidities known to be independently associated with fatigue, including diagnosed malignancies and mental health disorders, were excluded. All clinical and laboratory data were prospectively recorded in the institutional electronic registry (Arthritis and Rheumatism Database and Information System). Ethical approval for the study protocol was obtained from the Ethics Committee of the Medical Faculty, Eberhard Karls University of Tuebingen, and the University Hospital Tuebingen (Project Number: 070/2024BO2). All participants (or their legal guardians) provided written informed consent prior to inclusion in the study.

### 2.1. Data and Assessments

Patient- and disease-related variables included biological sex, ethnicity, age at symptom onset, at diagnosis, and at treatment start. Follow-up intervals were captured. Disease-associated clinical manifestations and phenotypes were recorded. Laboratory parameters comprised inflammatory markers, including serum amyloid A (SAA) and C-reactive protein (CRP), as well as identified gene variants and their classification according to the American College of Medical Genetics and Genomics (ACMG) guidelines [15]. Therapy data were documented, including medication type, dosage, and administration frequency.

The baseline visit was defined as the initial patient assessment conducted prior to or at initiation of therapy; the last follow-up visit was defined as the final patient assessment during the study period. Disease duration was defined as the interval from symptom onset to the last clinical visit, and treatment delay as the interval from symptom onset to initiation of effective anti-inflammatory therapy. For the analysis, both timeframes were dichotomized into ≤10 years and >10 years.

### 2.2. Concepts, Definitions, and Instruments

(1) **Disease activity** was assessed using validated instruments, including the Physician Global Assessment (PGA) and the Patient/Parent Global Assessment (PPGA), with both quantifying disease activity along a 10 cm visual analog scale (VAS), where 0 indicated no disease activity and 10 represented maximal disease activity. Categories of clinical disease activity were determined by PGA on a 10 cm VAS and categorized as mild (<2), moderate (2–4), and high (>4), as described previously (REF). Complete remission was defined as PGA ≤ 2, with CRP ≤ 0.5 mg/dl and/or SAA ≤ 10 mg/L. Partial remission was PGA > 2 and ≤5, with CRP > 0.5 mg/dl but ≤3 mg/dl and/or SAA > 10 mg/L but ≤30 mg/L. Non-remission was defined as PGA > 5, with CRP > 3 mg/dl and/or SAA > 30 mg/L [14].

(2) **Fatigue** was evaluated and quantified using two approaches: (a) A 10 cm VAS embedded within the PedsQL Present Functioning Visual Analogue Scales questionnaire [16], which comprises six parameters, including fatigue, and is routinely completed by patients prior to their outpatient visit. A score of 0 indicated no fatigue, whereas a score of 10 indicated maximal fatigue. Fatigue was considered present if the VAS score was ≥1. (b) The PedsQL Multidimensional Fatigue Scale (MFS) [17], an 18-item questionnaire comprising three subscales—General Fatigue, Sleep/Rest Fatigue, and Cognitive Fatigue—scored on a 0–100 scale, with higher scores indicating lower fatigue and better functioning. For the statistical analyses, scores were reverse-transformed (100—original score) so that higher values consistently reflected greater fatigue burden. The instrument has been validated for both child self-report (ages 5–18 years) and parent proxy-report (ages 2–18 years) [17].

(3) **Depressive symptoms** were assessed using the Center for Epidemiologic Studies Depression Scale (CES-D) [11] and its revised version, the CESD-R [18]. Both are 20-item self-report questionnaires measuring depressive symptomatology in the general population. While the original CES-D employs a 0–3 scoring system, the CESD-R uses a 0–4 scale. In accordance with previously described and widely applied practice, responses scored as 4 on the CESD-R were recoded to 3 to facilitate comparability and simplify the assessment of clinical depression risk [18]. A cut-off score of ≥16 was applied to define clinically relevant depressive symptoms [19].

Disease activity (PGA/PPGA) and fatigue VAS from the PedsQL Present Functioning Scales were recorded at every visit. PedsQL-MFS and CES-D/CESD-R were obtained only at the last follow-up. Each patient, therefore, contributed multiple measurements for disease activity and fatigue VAS, and one measurement for PedsQL-MFS and depression.

### 2.3. Statistical Analysis

Continuous variables were presented as median (IQR) and categorical variables as counts and percentages. Group comparisons used the Mann–Whitney U test for continuous data and Chi-square or Fisher’s exact tests for categorical data. Paired baseline–last visit comparisons employed the Wilcoxon signed-rank test. Correlations were assessed using Spearman’s ρ for non-parametric and Pearson’s r for parametric data (|ρ|/|r| < 0.3 = weak, 0.3–0.5 = moderate, and >0.5 = strong). To identify independent determinants of fatigue, relevant variables including age group, pathogenicity of variants, disease duration, treatment delay, and depression risk were first tested in univariable analyses. Those showing significant associations were subsequently included in multivariable linear regression models. Analyses were conducted in IBM SPSS Statistics v28.0.1.1, with *p* < 0.05 considered statistically significant.

## 3. Study Endpoints

The primary endpoint was Overall Fatigue measured by the PedsQL-MFS at the last follow-up visit. Secondary endpoints, all assessed at the last follow-up visit, included the following: (1) PedsQL-MFS subdomain scores (General, Sleep/Rest, and Cognitive Fatigue), (2) fatigue assessed by VAS, and (3) depression scores measured by CES-D/CESD-R.

## 4. Results

### 4.1. Patient Characteristics

The cohort consisted of 66 individuals affected by auto-inflammatory diseases, with a balanced distribution regarding biological sex and age. CAPS and FMF were represented in similar proportions. Genetic testing revealed a heterogeneous spectrum of variants, including pathogenic mutations as well as variants of uncertain significance, while more than one-third of patients showed no identifiable variant. Most patients had a German background, with a smaller proportion of Turkish origin. An overview of the demographic and genetic characteristics is provided in Table 1.

### 4.2. Clinical Presentation

Symptoms generally began in early childhood, and treatment was initiated across a wide age range. During long-term follow-up, all patients achieved complete remission at the last visit. Most remained in remission while receiving anti-inflammatory therapy, most commonly colchicine or IL-1 inhibitors. Treatment and follow-up characteristics are presented in Table 1.

In FMF patients (n = 31), the most common findings were abdominal pain (97%) and febrile attacks (94%), followed by arthritis/arthralgia (48%), stress- or infection-triggered attacks (45%), myalgia (42%), headache (39%), irritability (36%), and lymphadenopathy (23%). All CAPS patients (n = 35) had flares triggered by stress, cold, or infection. Urticarial rash (89%), arthralgia (74%), headache and conjunctivitis (71%, respectively), and myalgia and irritability (66%, respectively) were frequently seen. Complications included hearing loss (40%), tinnitus (26%), amyloidosis and skeletal abnormalities (14%, respectively), and aseptic meningitis (6%).

### 4.3. Disease Activity

Baseline PGA and PPGA values were consistent with moderate disease activity and declined to zero at the final assessment, indicating complete disease inactivity. A statistically significant disparity in fatigue severity between CAPS and FMF was evident, as presented in Table 2. Adults showed a trend toward higher baseline PGA scores compared with children (median PGA 5 (range 3–9) vs. 3 (range 2–9), *p* = 0.08).

#### 4.3.1. Fatigue

**Figure 1:**
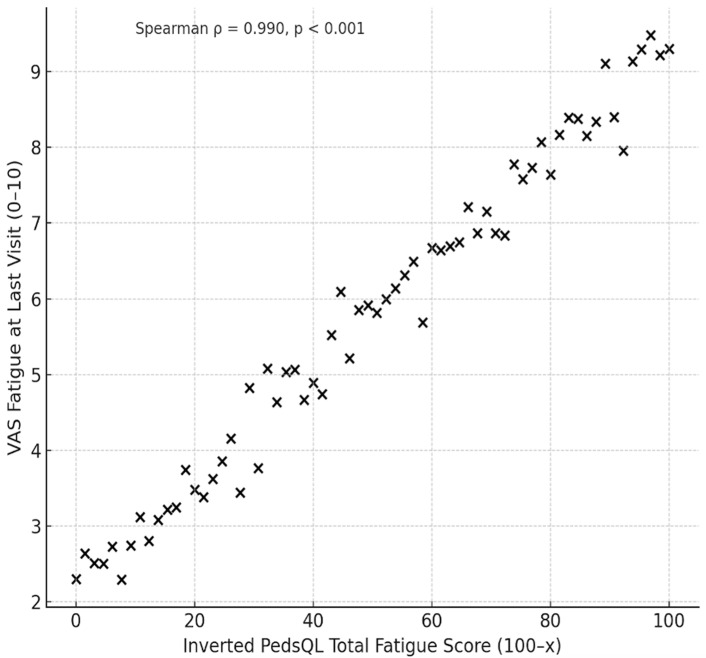
Correlation between inverted PedsQL Overall Fatigue Score (100–x) and VAS Fatigue score in patients with inactive auto-inflammatory disease. A strong positive correlation was observed between inverted PedsQL Overall Fatigue Score (100–x) and VAS Fatigue at the last visit (Spearman’s ρ = 0.942, *p* < 0.001).

At baseline, Overall Fatigue levels were high and remained within the moderate-to-high range at the last visit despite well-controlled disease activity. Inverted PedsQL-MFS scores indicated substantial impairment across all fatigue domains, with values markedly higher than those of the general population (Table 2). A significant correlation between the inverted PedsQL Overall Fatigue Score (100–x) and VAS Fatigue at the last visit was observed (Pearson’s r = 0.918, *p* < 0.001; Spearman’s ρ = 0.942, *p* < 0.001) (Figure 1).

In the comparison of CAPS and FMF patients, significant differences were confined to fatigue-related measures. CAPS patients demonstrated greater Cognitive Fatigue and substantially higher baseline VAS Fatigue scores. No additional between-group differences reached statistical significance, as detailed in Table 2.

#### 4.3.2. Depression

Depressive symptoms, as measured by CES-D/CESD-R, were generally elevated across the cohort, indicating a notable psychological burden in patients with inactive auto-inflammatory disease. Although CAPS patients tended to report slightly higher levels of depressive symptoms compared with FMF patients, this difference did not reach statistical significance (Table 2). Adults had slightly higher scores than children, with median values of 15 (0–42) versus 13 (0–45). This difference did not reach statistical significance (ρ = 0.244).

#### 4.3.3. Risk Factors Associated with Increased Fatigue

**Figure 2:**
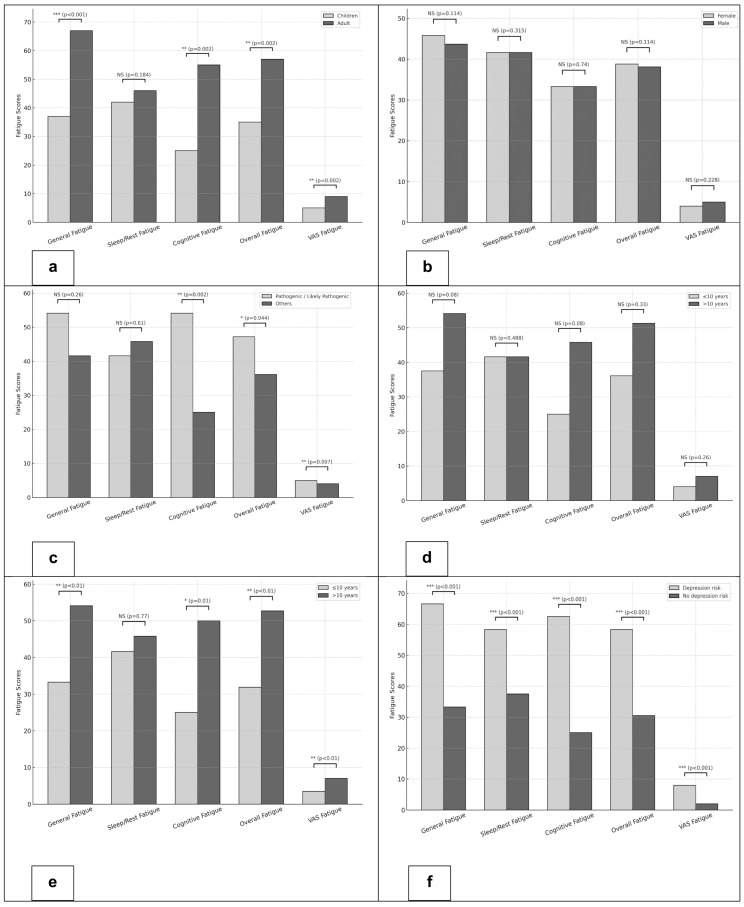
**Group comparisons of inverted PedsQL-MFS fatigue domain scores.** (**a**) **Children vs. adults:** Adults reported significantly higher scores in General, Cognitive, and Overall Fatigue, as well as VAS Fatigue (all *p* ≤ 0.002), while Sleep/Rest Fatigue did not differ between groups (*p* = 0.184). (**b**) **Biological sex:** No significant sex-related differences were observed across General, Sleep/Rest, Cognitive, Overall, or VAS Fatigue domains (all *p* > 0.05). (**c**) **Pathogenicity of the gene variant:** Cognitive, Overall, and VAS Fatigue were significantly higher in the pathogenic/likely pathogenic group (*p* = 0.002, *p* = 0.044, and *p* = 0.007, respectively), whereas General and Sleep/Rest Fatigue did not differ significantly between groups. (**d**) **Delay to effective treatment:** A trend toward higher fatigue was observed in patients with longer treatment delay (>10 years), but none of the differences reached statistical significance. (**e**) **Disease duration:** Patients with disease duration > 10 years exhibited significantly higher General, Cognitive, Overall, and VAS Fatigue scores, while Sleep/Rest Fatigue did not differ between groups. (**f**) **Depression:** After inversion of PedsQL-MFS scores (100–x), higher values reflect greater fatigue burden. Patients with depression risk (based on CES-D/CESD-R scores) showed significantly higher fatigue across all domains and VAS Fatigue (all *p* < 0.001). *: *p* < 0.05, **: *p* < 0.01, ***: *p* < 0.001.

**(1) Age:** Adults reported significantly higher fatigue scores across multiple domains compared to children. Median inverted PedsQL-MFS scores were 66.6 vs. 37.5 for General Fatigue (*p* < 0.001), 54.1 vs. 25.0 for Cognitive Fatigue (*p* = 0.002), and 56.9 vs. 34.7 for Overall Fatigue (*p* = 0.002). Similarly, the VAS Fatigue was significantly higher among adults (median 7 vs. 4; *p* = 0.002) (Figure 2a).

**(2) Biological sex:** No significant differences in fatigue were observed. Median inverted PedsQL-MFS scores for General, Sleep/Rest, Cognitive, and Overall Fatigue as well as fatigue VAS scores were comparable between females and males (Figure 2b).

**(3) Pathogenicity of the gene variant:** Patients with pathogenic/likely pathogenic variants exhibited significantly higher levels of fatigue compared to those with other or no variants. Median inverted PedsQL-MFS scores were 54.1 vs. 25.0 for Cognitive Fatigue (*p* = 0.002) and 47.2 vs. 36.1 for Overall Fatigue (*p* = 0.044). VAS Fatigue scores were also higher in the pathogenic/likely pathogenic group (median 5 vs. 4; *p* = 0.007) (Figure 2c).

**(4) Delay to effective treatment:** Patients with a treatment delay longer than 10 years tended to report higher fatigue levels. This trend was observed across PedsQL-MFS domains, particularly General and Cognitive Fatigue, as well as in VAS Fatigue (Figure 2d)

**(5) Disease duration:** Patients with a disease duration of more than 10 years reported significantly higher fatigue scores. Median inverted PedsQL-MFS scores were 54.1 vs. 33.3 for General Fatigue (*p* < 0.01), 50.0 vs. 25.0 for Cognitive Fatigue (*p* = 0.01), and 52.7 vs. 31.9 for Overall Fatigue (*p* < 0.01). Correspondingly, the fatigue VAS scores were higher in the group with longer disease duration (median 7 vs. 3.5; *p* < 0.01) (Figure 2e).

**(6) Depression:** Overall, 27 of 66 patients (41%) met the threshold for high depression risk, defined as a CES-D/CESD-R score ≥ 16. Patients with a high depression risk had markedly higher fatigue scores across all domains of the PedsQL-MFS compared with those without an elevated depression risk. Median scores were 66.6 vs. 33.3 for General Fatigue, 58.3 vs. 37.5 for Sleep/Rest Fatigue, 62.5 vs. 25.0 for Cognitive Fatigue, and 58.3 vs. 30.5 for Overall Fatigue (all *p* < 0.001). Consistently, VAS Fatigue scores were significantly higher in the high depression risk group (median 8 vs. 2; *p* < 0.001) (Figure 2f).

#### 4.3.4. Risk Model for Fatigue in Inactive AID

Depression consistently emerged as the strongest independent predictor of fatigue in multivariable regression analyses accounting for age group (adult vs. child), pathogenicity of the gene variant, delay to effective treatment, and disease duration. Depression risk was significantly associated with higher fatigue burden across all PedsQL domains and VAS Fatigue (all *p* < 0.001). Adult age was independently associated with greater Cognitive Fatigue (B = 15.7, *p* = 0.039), and disease duration showed a trend towards higher Sleep/Rest and General Fatigue (Table 3). The overall model explained 52% of the variance in fatigue (R^2^ = 0.52).

## 5. Discussion

This study provided a systematic evaluation of fatigue and depression in patients with inactive auto-inflammatory diseases. Despite controlled disease activity, fatigue scores were significantly elevated, reflecting the ongoing burden. Importantly, disease-specific impacts on the distinct fatigue domains such as Cognitive Fatigue in CAPS patients were documented. The demonstrated strong correlation between VAS Fatigue and PedsQL-MFS highlights the clinical utility of both measures in capturing highly relevant patient-reported outcomes. Depression emerged as the strongest independent determinant of fatigue. Other putative risk factors such as age, disease duration, and pathogenic variants were found to be less relevant in well-controlled auto-inflammatory disease. These findings underscore the need for a precision health approach that addresses fatigue as a distinct and clinically relevant burden in auto-inflammatory diseases, beyond the control of inflammatory disease activity, to prevent overtreatment and ensure optimal patient care.

Fatigue persisted in auto-inflammatory diseases despite adequate disease control. Our findings demonstrate consistently higher levels of fatigue across all domains in inactive disease compared with the general population [20]. Distinct disease-related fatigue patterns were identified. CAPS patients reported significantly higher Cognitive Fatigue than those with FMF, supporting the critical biological role of the central nervous system as a disease target in CAPS—including aseptic meningitis, brain atrophy, and sensorineural hearing loss [2]—and contributing to attentional and memory-related impairment. Fatigue needs to be explored and addressed at all stages of disease including its distinct domains to enable targeted strategies through precision health approaches. Taken together, these findings highlight the importance of comprehensive diagnostic and therapeutic strategies to adequately address fatigue in auto-inflammatory diseases.

Depression emerged as the strongest independent determinant of fatigue in patients with inactive AID. Factors such as adult age, longer disease duration and pathogenic variants were also found to be associated with higher fatigue in univariable analyses; however, their explanatory power diminished in multivariable models. Meta-analyses in rheumatoid arthritis and systemic lupus erythematosus have demonstrated a high prevalence of depression and strong associations with greater fatigue, impaired quality of life, and reduced adherence to therapy [7,21,22]. While an inflammatory etiology of depression has been proposed [9], depression in inactive inflammation remains poorly understood. In pediatric cohorts, depression and fatigue often remain underdiagnosed; nevertheless, their presence strongly predicted impaired school attendance, reduced social participation, and adverse developmental outcomes [8]. Our findings indicate that depressive symptoms constitute a major determinant of fatigue in auto-inflammatory diseases, independent of underlying inflammatory activity. This highlights the importance of integrating the assessment and management of modifiable psychological factors into routine clinical care. Multivariable modeling further suggests that, beyond depression, additional factors such as psychosocial stressors or sleep-related disturbances may also contribute to the Overall Fatigue burden.

There were several limitations to this study. The small sample size from a single center limits its generalizability. Socioeconomic factors and life circumstances, as well as their impact, were not fully captured; however, the data reflect a real-life cohort within a generally well-supported health and social care system in Germany. In addition, a contemporaneous healthy control group was lacking; however, large normative population data for the PedsQL-MFS [20] provided validated comparators.

Given the substantial burden of fatigue and its close link to depressive symptoms, targeted and multidimensional management approaches are warranted. Early psychological assessment, evidence-based psychotherapeutic interventions, optimization of anti-inflammatory treatment, structured physical activity, and coordinated care between rheumatology and mental health services may help address both fatigue and depressive comorbidities. Routine screening and stepped-care strategies could further facilitate timely identification and individualized management in this population.

## 6. Conclusions

Fatigue is a key symptom of active and inactive auto-inflammatory disease, significantly contributing to its individual and societal burden. Depression was found to be the strongest risk factor for debilitating fatigue in patients with inactive disease. There is an urgent need for diagnostic and therapeutic precision health approaches for depression and fatigue when caring for children and adults living with auto-inflammatory diseases. These data emphasize the urgent need for systematic screening and integrated approaches targeting both inflammatory and psychological domains of disease.

## Figures and Tables

**Table 1:** Baseline characteristics of consecutive pediatric and adult patients with inactive auto-inflammatory disease.

Characteristic	
**Total patients**	66
**Biological sex, n (%)**	
Female	34 (52)
Male	32 (48)
**Age group, n (%)**	
Pediatric (<18 years)	39 (59)
Adult (≥18 years)	27 (41)
**Diagnosis, n (%)**	
CAPS	35 (53)
FMF	31 (47)
**Pathogenicity of gene variant**, **n (%)**	
Pathogenic/likely pathogenic variant	27 (41)
Variant of uncertain significance (VUS)	15 (23)
No variant detected	24 (36)
**Ethnicity, n (%)**	
German	52 (79)
Turkish	14 (21)
**Disease status at last visit, n (%)**	
Clinical remission on medication (CRM)	61 (93)
Clinical remission off medication (CR)	5 (7)
**Treatment, n (%)**	
Colchicine	25 (38)
Canakinumab	32 (49)
Anakinra	4 (6)
**Age at symptom onset (years), median (IQR)**	4 (2–8)
**Age at treatment start (years), median (IQR)**	8 (5–14)
**Follow-up time (years), median (IQR)**	7 (2–20)

**Table 2:** Comparison of disease activity, fatigue, and depression between cohorts of patients with auto-inflammatory diseases.

	Total Cohort N = 66	CAPS N = 35	FMF N = 31	Comparison CAPS/FMF
**Disease Activity**				
Median PGA at first visit (IQR) ^†^	4.00 (3–6)	4.00 (3–6)	4.00 (3–5)	0.386
Median PPGA at first visit (IQR) ^†^	4.00 (3–5)	4.00 (3–5)	4.00 (2–5)	0.203
**Fatigue**				
Median VAS Fatigue at first visit (IQR) ^†^	6.00 (5–8)	8.00 (5–9)	5.00 (4–6)	<0.001 *
Median VAS Fatigue at last visit (IQR) ^†^	4.50 (1–7)	5.00 (1–8)	4.00 (0–6)	0.107
Median PedsQL—General Fatigue at last visit (IQR) ^‡^	45.8 (20–64)	45.8 (25–75)	41.6 (10–54)	0.078
Median PedsQL—Sleep/Rest Fatigue at last visit (IQR) ^‡^	41.6 (29–58)	41.6 (33–58)	45.8 (24–58)	0.887
Median PedsQL—Cognitive Fatigue at last visit (IQR) ^‡^	33.3 (17–59)	37.5 (25–67)	25 (11–50)	0.040 *
Median PedsQL—Overall Fatigue at last visit (IQR) ^‡^	38.8 (26–58)	40.2 (26–61)	36.1 (16–55)	0.192
**Depression**				
Median Depression score (CES-D/CESD-R overall) at last visit (IQR) ^§^	13.00 (10–23)	14.00 (10–27)	12.00 (9–19)	0.280

Legend: PedsQL-MFS scores were inverted (100–x) for consistency with VAS Fatigue so that higher scores indicate greater fatigue. ^†^: Range for PGA, PPGA, and VAS Fatigue scores: 0–10 cm. ^‡^: Range for inverted PedsQL Overall Fatigue Score and subscales: 0–100. ^§^: Range for CES-D/CESD-R: 0–60. Values are presented as median (IQR). IQR = interquartile range. *: *p* < 0.05.

**Table 3:** Risk profile of Overall Fatigue and distinct domains of fatigue in patients with inactive auto-inflammatory diseases—multivariable regression analyses.

	Significant Predictors	B (95% CI)	*p*-Value
**Risk Model for Overall Fatigue**			
**Inverted PedsQL-MFS Score**	Depression risk	27.0 (18.8–35.2)	**<0.001**
**Risk Model for Sleep/Rest Fatigue**			
**Inverted PedsQL Sleep/Rest Fatigue Score**	Depression risk	21.5 (11.5–31.6)	**<0.001**
	Disease duration (trend)	9.9 (–0.4–20.3)	0.060
**Risk Model for Cognitive Fatigue**			
**Inverted PedsQL Cognitive Fatigue Score**	Depression risk	28.6 (18.3–38.9)	**<0.001**
	Adult vs. child	15.7 (0.9–30.6)	0.039
**Risk Model for General Fatigue**			
**Inverted PedsQL General Fatigue Score**	Depression risk	31.0 (22.4–39.6)	**<0.001**
	Disease duration (trend)	–8.8 (–17.7–0.1)	0.053
**Risk Model for VAS Fatigue Score**			
**VAS Fatigue Score**	Depression risk	3.5 (2.2–4.8)	**<0.001**
	Disease duration (trend)	1.7 (–0.2–3.7)	0.081

Legend: The multivariable regression models were constructed to identify independent determinants of fatigue. All models were adjusted for age group (adult vs. child), pathogenicity of variants, disease duration, treatment delay, and depression risk.

## Data Availability

Upon reasonable request and with obtained ethical approval, the dataset can be made available by the corresponding author.
